# Human Umbilical Cord Blood-Derived Serum for Culturing the Supportive Feeder Cells of Human Pluripotent Stem Cell Lines

**DOI:** 10.1155/2016/4626048

**Published:** 2015-12-29

**Authors:** Ruttachuk Rungsiwiwut, Praewphan Ingrungruanglert, Pranee Numchaisrika, Pramuan Virutamasen, Tatsanee Phermthai, Kamthorn Pruksananonda

**Affiliations:** ^1^Human Embryonic Stem Cell Research Center, Reproductive Medicine Unit, Department of Obstetrics and Gynecology, Faculty of Medicine, Chulalongkorn University, Bangkok 10330, Thailand; ^2^Stem Cell and Cell Therapy Research Unit, Faculty of Medicine, Chulalongkorn University, Bangkok 10330, Thailand; ^3^Stem Cell Research and Development Unit, Department of Obstetrics and Gynecology, Faculty of Medicine Siriraj Hospital, Mahidol University, Bangkok 10700, Thailand

## Abstract

Although human pluripotent stem cells (hPSCs) can proliferate robustly on the feeder-free culture system, genetic instability of hPSCs has been reported in such environment. Alternatively, feeder cells enable hPSCs to maintain their pluripotency. The feeder cells are usually grown in a culture medium containing fetal bovine serum (FBS) prior to coculture with hPSCs. The use of FBS might limit the clinical application of hPSCs. Recently, human cord blood-derived serum (hUCS) showed a positive effect on culture of mesenchymal stem cells. It is interesting to test whether hUCS can be used for culture of feeder cells of hPSCs. This study was aimed to replace FBS with hUCS for culturing the human foreskin fibroblasts (HFFs) prior to feeder cell preparation. The results showed that HFFs cultured in hUCS-containing medium (HFF-hUCS) displayed fibroblastic features, high proliferation rates, short population doubling times, and normal karyotypes after prolonged culture. Inactivated HFF-hUCS expressed important genes, including Activin A, FGF2, and TGF*β*1, which have been implicated in the maintenance of hPSC pluripotency. Moreover, hPSC lines maintained pluripotency, differentiation capacities, and karyotypic stability after being cocultured for extended period with inactivated HFF-hUCS. Therefore, the results demonstrated the benefit of hUCS for hPSCs culture system.

## 1. Introduction

Human pluripotent stem cells (hPSCs) can be generated from isolation of the pluripotent cells using preimplantation embryos, called “human embryonic stem cells (hESCs)” [1] or the reprogramming of somatic cells using exogenous genes, resulting in “human induced pluripotent stem cells (hiPSCs)” [2]. Both hESCs and hiPSCs share phenotypic and molecular genetic similarities [3]. hPSCs can be used as a model for the study of developmental biology, toxicity, and cell-based therapy [4]. Conventionally, hPSCs are derived and propagated through coculture with supportive feeder cells isolated from mice [1, 2]. Feeder cells play an important role in supporting the pluripotency of hPSCs by producing a complex microenvironment for the growth of hPSCs. Feeder cells secrete growth factors, such as FGF2, TGF-*β*1, or Activin A, involving the specific pathway controlling the pluripotency of hPSCs. Moreover, extracellular matrices, such as laminin, fibronectin, or collagen, produced from the feeders, are necessary for cell interaction, cell migration, or cell proliferation [5].

For therapeutic purposes, avoiding the cross-contamination of animal pathogens to hPSCs through the replacement of mouse feeder cells with the feeder cells derived from human tissue should be considered. Human feeder cells, such as human foreskin fibroblasts (HFFs), human mesenchymal stem cells (hMSCs), human amniotic epithelial cells (hAECs), or human fallopian tube-derived cells, were used as supportive feeder cells for the culture of hPSCs [6]. Moreover, previous studies have demonstrated the possibility to generate genetically modified human feeder cells for the long-term support of hPSCs [7, 8]. For routine culture, human cells were cultured in medium supplemented with fetal bovine serum (FBS). Although FBS promotes human cell proliferation, human cells bare the risk of contamination of bovine viruses or other pathogens. For clinical purposes, the use of animal products in human cell culture should be avoided. Since human serum (HS) shows a positive effect on culture of mesenchymal stem cells, it appears to be a promising candidate for FBS replacement. The previous studies demonstrated that HS can be used for culturing the supportive feeder cells of hESCs [9, 10] and hMSCs showed greater proliferation in the HS-containing medium compared with FBS-containing medium [11, 12]. Nonetheless, HS not only promoted hMSC cell proliferation but also enhanced osteogenic differentiation [13]. Due to the progressive research for the therapeutic application of hPSCs, it is necessary to develop xeno-free culture conditions for the maintenance of hPSCs. Recent studies have shown that both feeder cells and hPSCS can be generated and cultured under the good manufacturing practice (GMP) [14, 15] as a useful step forward for the application of hPSCs in the field of regenerative medicine. Recently, human cord blood-derived serum (hUCS) showed a positive effect on culture of mesenchymal stem cells [16, 17]. Therefore, it is interesting to test whether hUCS can be used for culture of feeder cells of hPSCs.

The objective of the present study was to compare (i) growth, proliferation, and karyotypic stability of HFFs when cultured in a medium containing hUCS (HFF-hUCS) versus FBS (HFF-FBS), (ii) the characteristics of inactivated HFF-hUCS and inactivated HFF-FBS, and (iii) the pluripotent characteristics of hPSCs after having been cocultured for a long period with inactivated HFF-hUCS and inactivated HFF-FBS.

## 2. Results

### 2.1. Effect of Serum Supplementation on the Morphology and Proliferation of Human Foreskin Fibroblasts

**Figure 1:**
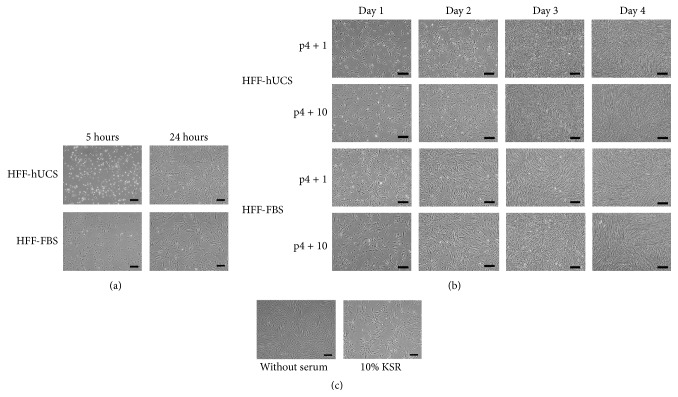
Effect of serum supplementation on the growth kinetics of human foreskin fibroblasts. Human foreskin fibroblasts were enzymatically dissociated and plated onto culture dishes. HFFs were cultured in the media containing human cord blood-derived serum (hUCS; HFF-hUCS), fetal bovine serum (FBS; HFF-FBS), knockout serum replacement (KSR) or without serum supplementation. Dissociated cells were observed for attachment behavior and growth kinetics. Differences in cell attachment between HFF-hUCS and HFF-FBS were observed. At 5 hours after plating, nearly all of the dissociated HFF-FBS were attached to the surface of the culture dish and exhibited a fibroblastic-like morphology, while most of HFF-hUCS remained suspended in the culture medium. At 24 hours after plating, all HFF-FBS and HFF-hUCS were attached to the culture dishes and displayed typical fibroblast cell morphology (a). Similar proliferation patterns between HFF-hUCS and HFF-FBS were observed. HFF-hUCS and HFF-FBS progressively proliferated and reached confluency at 3 to 4 days after seeding. The proliferation behavior at early passage (p4 + 1) was similar to that at intermediate passage (p4 + 10) (b). In contrast to HFF-hUCS and HFF-FBS, HFFs cultured in the medium containing 10% KSR and without serum supplementation could not reach the confluency even being cultured for 7 days (c). HFF = human foreskin fibroblasts, FBS = fetal bovine serum, hUCS = human cord blood-derived serum, KSR = knockout serum replacement, and p = passage number. Scale bars = 200 *μ*m.

The HFFs were cultured in medium containing either hUCS or FBS, and the morphology and growth were subsequently observed. We first observed the difference of cell attachment between the two culture media. At 5 hours after dissociation, nearly all dissociated HFF-FBS were attached to the surface of the culture dish. HFFs were transformed from a round to a fibroblastic-like shape. In contrast, most HFF-hUCS remained suspended in the culture medium. However, at 24 hours after dissociation, there were no notable differences in cell attachment and morphology between HFF-FBS and HFF-hUCS and most of the cells were attached to the culture dishes and displayed typical fibroblast cell morphology (Figure 1(a)).

After seeding the cells, the growth of HFF-hUCS and HFF-FBS was continuously observed until reaching confluency. We observed that HFFs cultured in both media progressively proliferated and reached confluency between 3 and 4 days after seeding the cells. The growth behaviors were similar at both early (p4 + 1) and intermediate passage (p4 + 10) (Figure 1(b)). In contrast, HFFs cultured in the medium without serum supplementation and in the medium supplemented with 10% KSR attached to the culture dishes but the cells could not reach confluency even being cultured for 7 days (Figure 1(c)).

**Figure 2:**
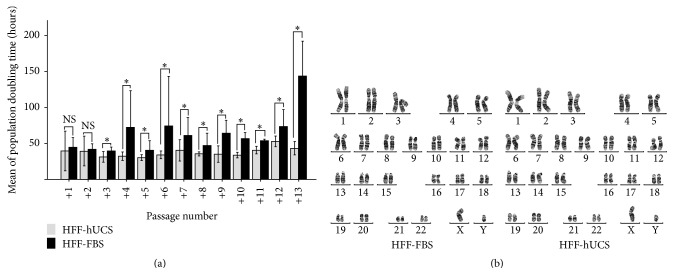
Effect of serum supplementation on cell proliferation and karyotype stability of human foreskin fibroblasts. The cell proliferation of human foreskin fibroblasts (HFFs) was evaluated by determination of their population doubling time (PDT). The ability of HFFs to maintain their normal karyotype was assessed by the G-banding method. The PDT of HFFs cultured in the media containing human cord blood-derived serum (hUCS; HFF-hUCS) and fetal bovine serum (FBS; HFF-FBS) from p4 + 1 to p4 + 13 were determined. The HFF-hUCS from p4 + 3 to p4 + 13 displayed a significantly shorter (*P* < 0.05) PDT than HFF-FBS (a). The karyotype analysis of HFF-FBS and HFF-hUCS demonstrated that both HFFs cultured in hUCS- or FBS-containing media maintained a normal 46,XY karyotype after prolonged culture (b). Error bars represent the standard error of mean (SEM). HFF = human foreskin fibroblasts, FBS = fetal bovine serum, hUCS = human cord blood-derived serum, and NS = not significant. Scale bars = 200 *μ*m, ^*∗*^ *P* < 0.05.

Although the growth behavior of HFF-hUCS and HFF-FBS was not notably different, we observed some differences in the proliferation kinetics. The proliferation kinetics of HFF-hUCS and HFF-FBS were determined as the population doubling time (PDT) during continuous culture for up to 13 passages (p4 + 13). At passage numbers 4 + 3 to 4 + 13, HFF-hUCS displayed a significant shorter (*P* < 0.05) PDT compared with HFF-FBS (Figure 2(a)). The PDT of HFFs cultured in the medium without serum supplementation and supplemented with 10% KSR were not able to determine due to their proliferation insufficiency.

### 2.2. Karyotype Analysis of Human Foreskin Fibroblasts after Long-Term Culture in the Culture Medium Containing Human Umbilical Cord Blood-Derived Serum

The major effect of serum supplementation was observed in the proliferation of HFFs, showing that hUCS promotes better HFF proliferation than FBS. However, prior to using HFFs as feeder cells for culturing human pluripotent stem cells (hPSCs), we examined the genetic stability of HFFs through karyotype analysis of HFF-hUCS and HFF-FBS at p4 + 13 using the G-banding method. The results showed that culturing HFFs in hUCS-containing medium did not alter the karyotype of these cells. After culturing in either hUCS- or FBS-containing medium HFFs maintained a normal karyotype of 46,XY (Figure 2(b)).

### 2.3. Effect of Serum Supplementation on the Morphology and Gene Expression of Inactivated Human Foreskin Fibroblast Feeder Cells

**Figure 3:**
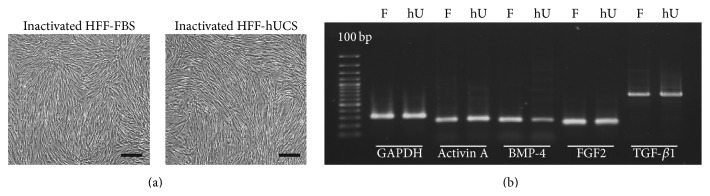
Morphology and gene expression of inactivated human foreskin fibroblasts prior to use as the feeder layer. HFF-hUCS and HFF-FBS survived inactivation through mitomycin-C treatment and maintained typical fibroblast-like features (a). RT-PCR was performed for the detection of supportive feeder cell-related genes. Inactivated HFF-hUCS and inactivated HFF-FBS expressed Activin A, FGF2, TGF-*β*1, and BMP-4. GAPDH was used as the house keeping gene (b). BMP = bone morphogenetic protein, FGF = fibroblast growth factors, TGF = transforming growth factor, HFF = human foreskin fibroblasts, F and FBS = fetal bovine serum, hU and hUCS = human cord blood-derived serum, RT-PCR = reverse transcription polymerase chain reaction, and GAPDH = glyceraldehyde 3-phosphate dehydrogenase. Scale bars = 200 *μ*m.

In the present study, we used HFF-hUCS and HFF-FBS between p4 + 5 and p4 + 10 to prepare feeder layer. After mitomycin C-inactivation, HFF-hUCS and HFF-FBS displayed typical fibroblast features (Figure 3(a)). Inactivated HFF-hUCS and inactivated HFF-FBS were cultured in hPSC culture media for 24 hours, and total RNA were collected and subjected to gene expression analysis using RT-PCR. As shown in Figure 3(b), inactivated HFF-hUCS and inactivated HFF-FBS expressed Activin A, FGF2, TGF-*β*1, and BMP-4, which have been implicated in the maintenance of hPSC pluripotency.

### 2.4. Human Pluripotent Stem Cells Maintained Pluripotency after Coculture with Inactivated HFF-hUCS Feeder Cells

**Figure 4:**
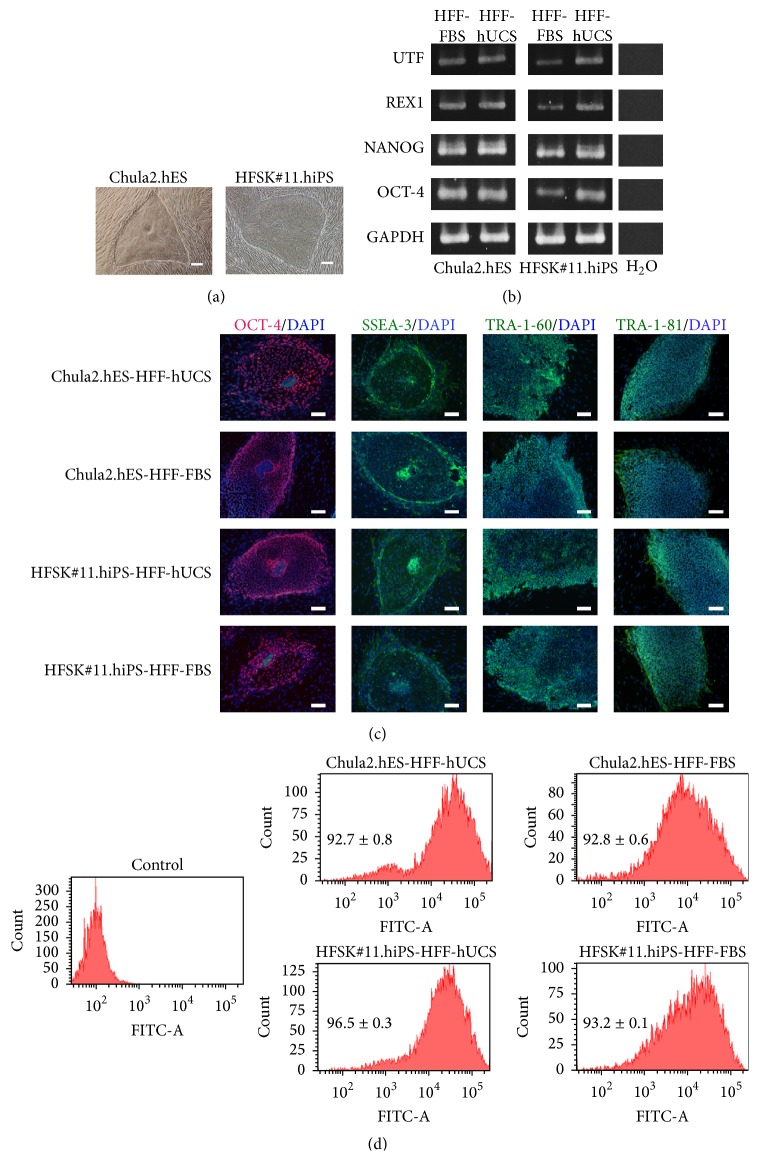
Characterization of human pluripotent stem cells after coculture with HFF-hUCS feeder cells. Two karyotypic normal hPSC lines, hESC (Chula2.hES) and hiPSC (HFSK#11.hiPS), were cocultured with inactivated HFF-hUCS or inactivated HFF-FBS, and subsequently the morphological appearance, pluripotent gene expression, and pluripotent marker expression were examined. hPSC lines showed undifferentiated colonies with defined boundaries and distinct prominent nuclei (a). hPSCs cocultured with inactivated HFF-hUCS expressed pluripotent genes, including OCT-4, NANOG, REX1, and UTF, similar to hPSCs cocultured with inactivated HFF-FBS (b). The pluripotent markers, including SSEA-3, TRA-1-60, TRA-1-81, and OCT-4, were detected in hPSCs cocultured with inactivated HFF-hUCS and inactivated HFF-FBS (c). The quantitative analysis of SSEA-4 expression through flow cytometry demonstrated that the percentage of SSEA-4-positive cells was not significantly different between hESCs cocultured with inactivated HFF-FBS and inactivated HFF-hUCS (92.8 ± 0.6 versus 92.7 ± 0.8, resp.). The percentage of SSEA-4-positive hiPSCs cocultured with inactivated HFF-hUCS (96.5 ± 0.3) was significantly higher than that for hiPSCs cocultured with inactivated HFF-FBS (93.2 ± 0.1) (*P* < 0.05; (d)). HFF = human foreskin fibroblasts, FBS = fetal bovine serum, hUCS = human cord blood-derived serum, and DAPI = 4′,6-diamidino-2-phenylindole. Scale bars = 200 *μ*m.

In the present study, karyotypic normal hPSC lines, including Chula2.hES and HFSK#11.hiPS, were used. Prior to examining the pluripotency of hPSCs, the cells were continuously grown on inactivated HFF-FBS or HFF-hUSC feeder cells for at least 10 passages. The morphology of hPSCs, when cultured on inactivated HFF-hUCS feeder cells, was not notably different from that of cells cultured on inactivated HFF-FBS feeder cells. Both hPSC lines showed undifferentiated colonies with defined boundaries and distinct prominent nuclei (Figure 4(a)).

hPSCs cultured on inactivated HFF-hUCS and inactivated HFF-FBS feeder cells expressed pluripotent genes, including OCT-4, NANOG, REX1, and UTF (Figure 4(b)). Immunostaining demonstrated that hPSCs cultured on inactivated HFF-hUCS and inactivated HFF-FBS feeder cells expressed pluripotent markers, including SSEA-3, TRA-1-60, TRA-1-81, and OCT-4 (Figure 4(c)). In addition, the quantitative analysis of SSEA-4 expression using flow cytometry demonstrated that the percentage of SSEA-4 positive cells between hESCs cocultured with inactivated HFF-FBS and inactivated HFF-hUCS was not significantly different (92.8 ± 0.6 versus 92.7 ± 0.8, resp.). Interestingly, the percentage of SSEA-4-positive hiPSCs cocultured with inactivated HFF-hUCS (96.5 ± 0.3) was significantly higher than that of hiPSCs cocultured with inactivated HFF-FBS (93.2 ± 0.1) (*P* < 0.05) (Figure 4(d)).

### 2.5. Differentiation Ability and Karyotypic Stability of Human Pluripotent Stem Cells

**Figure 5:**
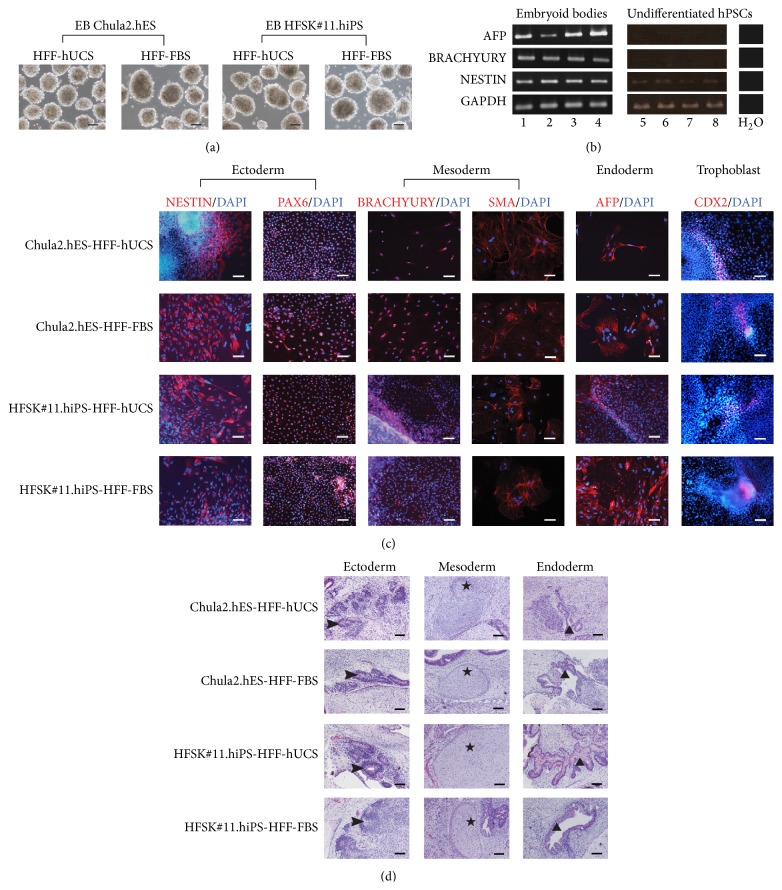
In vitro and in vivo differentiation of human pluripotent stem cell lines cocultured with inactivated HFF-hUCS. The differentiation capacities of hPSC lines cocultured with inactivated HFF-hUCS and inactivated HFF-FBS were determined through the formation of embryoid bodies (EBs) and teratoma formation. hPSC lines cocultured with inactivated HFF-hUCS formed EBs in suspension culture similar to hPSC lines cocultured with inactivated HFF-FBS (a). After the EBs were plated into culture dishes and continuously cultured for 21 days, the EBs differentiated to embryonic germ layers, including the ectoderm (NESTIN, PAX6), mesoderm (BRACHYURY, SMA), endoderm (AFP), and the trophoblast markers (CDX2) were detected through immunostaining (b) and RT-PCR (c). hPSCs lines cocultured with inactivated HFF-hUCS formed teratoma tissue and differentiated into ectoderm (neural rosette-like structure; arrowhead), mesoderm (cartilage; star), and endoderm (gut-like structure; triangle) similar to those cocultured with inactivated HFF-FBS (d). AFP = alpha-fetoprotein, smooth muscle actin (SMA), HFF = human foreskin fibroblasts, FBS = fetal bovine serum, hUCS = human cord blood-derived serum, hPSCs = human pluripotent stem cells, DAPI = 4′,6-diamidino-2-phenylindole, and RT-PCR = reverse transcription polymerase chain reaction. Lane 1 = EB Chula2.hES-HFF-hUCS, Lane 2 = EB Chula2.hES-HFF-FBS, Lane 3 = EB HFSK#11.hiPS-HFF-hUCS, Lane 4 = EB HFSK#11.hiPS-HFF-FBS, Lane 5 = undifferentiated Chula2.hES-HFF-hUCS, Lane 6 = undifferentiated Chula2.hES-HFF-FBS, Lane 7 = undifferentiated HFSK#11.hiPS-HFF-hUCS, and Lane 8 = undifferentiated HFSK#11.hiPS-HFF-FBS. Scale bars (a) and (d) = 200 *μ*m and (b) = 40 *μ*m.

The differentiation of hPSCs cultured on inactivated HFF-hUCS and inactivated HFF-FBS was confirmed based on embryoid body (EB) formation subsequent to differentiation in vitro. The results showed that hPSCs cultured on inactivated HFF-hUCS and inactivated HFF-FBS feeder cells formed three-dimensional EBs in suspension culture (Figure 5(a)). The in vitro differentiation of EBs toward three embryonic germ layers was confirmed by using immunostaining for ectoderm (NESTIN, PAX6), mesoderm (BRACHYURY, SMA), and endoderm (AFP). The RT-PCR results also confirmed the differentiation abilities of EBs toward ectoderm, (NESTIN), mesoderm (BRACHYURY), and endoderm (AFP). Moreover CDX2, a trophoblast marker, was also detected in EBs (Figures 5(b) and 5(c)).

In order to evaluate the in vivo differentiation of hPSCs, the cells were allowed to grow and differentiate after injection into immunodeficient mice. The teratoma formation assay was performed for in vivo differentiation test. The presence of structures that resemble the ectodermal, endodermal, and mesodermal tissues in the teratoma confirmed the differentiation capacities of hPSCs after being cocultured with inactivated HFF-hUCS and inactivated HFF-FBS (Figure 5(d)).

**Figure 6:**
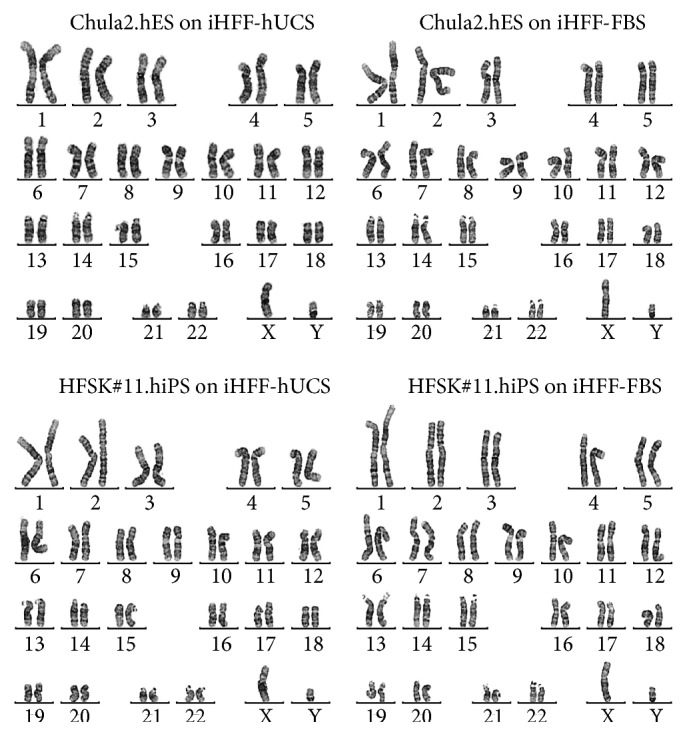
Karyotypic analysis of human pluripotent stem cell lines cocultured with inactivated HFF-hUCS. The karyotypic stability of hPSCs after prolonged coculture with inactivated HFF-hUCS was detected using the G-banding method. hPSC lines cocultured with inactivated HFF-hUCS maintained a normal karyotype similar to hPSCs lines cocultured with inactivated HFF-FBS. Chula2.hES maintained the 46,XY karyotype and HFSK#11.hiPS maintained the 46,XY karyotype on both types of feeder cells. iHFF-hUCS = inactivated HFF-hUCS and iHFF-FBS = inactivated HFF-FBS.

After coculturing hPSCs with inactivated HFF-hUCS and inactivated HFF-FBS for more than 10 passages, hPSCs were subjected to karyotype analysis. The results demonstrated that the coculture of diploid hPSC lines with inactivated HFF-hUCS feeder cells did not change the karyotype stability of these cells. Chula2.hES maintained the 46,XY karyotype and HFSK#11.hiPS maintained the 46,XY karyotype on both types of feeder cells (Figure 6).

## 3. Discussion

The successful derivation of human pluripotent stem cells (hPSCs), including human embryonic stem cells (hESCs) [1] and human induced pluripotent stem cells (hiPSCs) [2], is promising not only for use in the treatment of patients suffering from cell or tissue damage but also for drug discovery and the exploration of human developmental biology. hESCs can be derived from the preimplantation embryos, while hiPSCs can be generated through the reprogramming of somatic cells to the embryonic stem cell-like stage. Both hESCs and hiPSCs were conventionally derived and cocultured with mouse embryonic fibroblasts (MEFs) [1, 2]. Due to the progress of using hPSCs derivatives for therapeutic development program, the production of clinical grade hPSCs is needed. Therefore, it is important to develop a culture system being practical, simple, and clinically relevant for the generation and propagation of hPSCs. Although the feeder-free culture system is the ideal culture condition for derivation and culture of clinical grade hPSCs, this culture system is still facing some limitations. Firstly, many studies reported the successful derivation and culture of hPSCs under feeder-free culture system using Matrigel instead of feeder layer, resulting in robust proliferation of hPSCs. However, Matrigel is extracellular matrices isolated from mice, which makes them unsuitable for use as extracellular matrices for culturing clinical grade hPSCs. Secondly, the more relevant extracellular matrices originated from human recombinant proteins, including vitronectin, fibronectin, and laminin and their combinations have been shown to support the pluripotency of hPSCs. However, large-scale purification or production of biologically functional human proteins by recombination technologies is laborious and expensive [18]. Thirdly, the ability of feeder-free culture system to maintain the genetic stabilities of hPSCs remains controversial [19]. hPSC lines derived and/or cultured under the feeder-free conditions acquired karyotypic abnormalities during subsequent culture [19, 20].

On the other hand, the conventional culture system, using mouse embryonic fibroblasts (MEF) as the feeder cells, is still widely applied to ensure self-renewal and the pluripotency of hPSCs. MEFs play important roles in the regulation of hPSC pluripotency through the secretion of essential cytokines or molecules and extracellular matrices [21, 22]. Due to concerns about animal pathogens and immunogens using MEFs, several reports recommended the use of human fibroblasts, including human neonatal foreskin fibroblasts (HFFs) instead of MEFs [23–25]. The results demonstrated that HFFs showed the characteristics of supportive feeder cells for hPSCs. Interestingly, the current clinical trials using hPSCs to treat patients suffering from macular degeneration adopted the feeder culture system for propagation of hPSCs prior to differentiation of cells into the retinal-pigment epithelial (RPE) cells [26, 27]. The feeder cells appear thus to remain a good choice for coculture with hPSCs prior to use hPSCs for clinical treatment.

However, HFFs were routinely cultured and propagated in fetal bovine serum- (FBS-) containing medium prior to inactivation and used as the feeder cells for culturing hPSCs. FBS contains several unknown factors and is widely used for supplementation in animal and human cell culture media. The FBS supplementation in the culture medium influences the proliferation, maintenance, and differentiation of human stem cells [28, 29]. However, using FBS for culture of the HFFs might cause the contamination of bovine pathogen(s) to hPSCs, through the feeder cells. To reduce the cross contamination, FBS can be replaced with the human serum.

In the present study, we attempted to improve the culture conditions for hPSCs using human serum and human feeder cells. The human serum used in the present study was the human cord blood-derived serum (hUCS). hUCS has previously been demonstrated to have beneficial effects on the isolation, proliferation, and differentiation of human amniotic fluid stem cells (hAFS) and Wharton's jelly mesenchymal stromal cells by our collaborators [16, 17]. The commercial human foreskin fibroblasts (HFFs) used in the present study have been demonstrated as supportive feeder cells for the derivation of hESCs and hiPSCs [23–25]. Therefore, the aim of the present study was to examine the feasibility of using hUCS for culturing HFFs prior to the preparation of feeder cells and the subsequent use of inactivated HFFs for coculture with hPSCs. We first demonstrated that hUCS can efficiently replace FBS for the routine culture of HFFs. hUCS improved the proliferation of HFFs and maintained the karyotype and normal features of these cells. Secondly, mitomycin-C-inactivated HFFs, previously cultured with hUCS-containing media, expressed candidate genes, including Activin A, FGF2, and TGF-*β*, which are normally expressed by hPSC supportive feeder cells [21]. Thirdly, hPSC lines cocultured with inactivated HFFs, previously grown in hUCS-containing media, maintained pluripotency, differentiation, and a normal karyotype.

Serum contains factors that supply nutrients to the cells, protect cells from stress, and influence the attachment of almost all cell types. A major advantage of using human serum for culturing human cells, particularly human stem cells, is avoiding the contamination of animal pathogens to human cells. However, only the beneficial effects of using human serum in human stem cell culture have been demonstrated [11–13]. Under the culture conditions used in the present study, we observed that the type of serum presented in the HFF culture medium affected the attachment of HFFs after enzymatic dissociation. Shortly after replating, HFFs in FBS-containing medium attached to the surface of the culture dishes, while HFFs in hUCS-containing medium remained afloat. However, after 24 hours, the HFFs in the hUCS-containing medium were attached to the surface and displayed a morphology similar to the HFFs in FBS-containing medium. The delayed attachment of HFF-hUCS to the surface of the culture dish likely reflects differences in the cytokines, growth factors, or extracellular matrices contained in hUCS and FBS [30, 31].

Interestingly, the source of the serum also affected the proliferation kinetic of HFFs. The population doubling time (PDT) of HFFs after culture in medium containing hUCS was shorter than that observed with FBS. Extending the PDT limits the life span and use of cells for these experiments. Thus, hUCS provided more suitable nutrients and a better physiological environment for HFF cell division compared with FBS. Previous studies have demonstrated that using human serum for culturing HFFs extends the passage number of cultured HFFs. Therefore, numerous HFF stocks can be obtained for feeder supply for hPSC culture [5, 9]. The chromosomal stability of the cells is one of the most important issues for the application of stem cells for cell therapy. During the routine culture of human stem cells, chromosome stability was regularly analyzed every 5–10 passages. Prolonged culture or changing the culture conditions might lead to the karyotype instability of the cultured cells [18, 30]. Interestingly, the results of the karyotype analysis in the present study showed that although the culture medium was supplemented with hUCS, this serum did not alter the karyotype of HFFs after prolonged culture. Taken together, hUCS not only enhances cell proliferation but also preserves the HFF karyotypic stability. Therefore, hUCS is suitable for HFF culture medium supplementation.

To use HFFs as the feeder cells for culturing hPSCs, the cell division of HFFs should be inactivated. The inactivation of HFFs can be performed through exposure to gamma rays or supplementation with mitomycin-C into the culture medium. Prolonged incubation with a high concentration of mitomycin-C might damage fibroblast cells [32]. In the present study, HFFs cultured in hUCS- and FBS-containing media were treated with a standard concentration of mitomycin-C for a standard incubation time [33]. The difference in the morphological appearance was not notably observed between inactivated HFFs cultured in hUCS- and FBS-containing medium. HFFs cultured in hUCS were resistant and survived mitomycin C treatment, similar to the HFFs cultured in FBS. It has been previously demonstrated that the differences in the secretion of growth factors, cytokines, and extracellular matrices (ECM) distinguish supportive from nonsupportive feeder cells [21]. A proteomics approach is typically used to characterize the composition and interaction networks between the ECMs that support the maintenance of hESCs [34, 35]. In addition, the detection of the expression of supportive feeder-related genes, such as FGF2, Activin A, or TGF-*β*1, could also be used to examine supportive feeders prior to use for culturing hPSCs. In the present study, inactivated HFFs-hUCS and HFFs-FBS similarly expressed the selected genes, including Activin A, FGF2, and TGF-*β*1, which is typically detected in supportive feeder cells. These results were consistent with those of Eiselleova and colleagues [21]. The genes expressed from inactivated HFFs provided a complex network for the maintenance of hPSCs in the pluripotent state, suggesting that the supplementation of hUCS in the culture media might not change the supportive feeder cell nature of HFFs.

We further tested the pluripotency of hPSC lines after prolonged coculture with inactivated HFF-hUCS or inactivated HFF-FBS. The hPSC lines were cocultured with inactivated HFF in the serum-free culture medium. The serum-free culture medium is considered as the suitable culture medium for maintenance of hPSCs in order to use hPSCs for cell-based therapy. The results demonstrated that hPSC lines cocultured with inactivated HFF-hUCS feeders maintained pluripotency with normal hPSC colony morphology and the expression of pluripotent transcriptional factors, including OCT-4, NANOG, REX1, and UTF, and pluripotent markers, including SSEA-3, TRA-1-60, TRA-1-81, and OCT-4. Moreover, the quantified results of SSEA-4 expression, determined through flow cytometry, demonstrated that inactivated HFF-hUCS effectively support the pluripotency of hPSCs in a manner similar to inactivated HFF-FBS. In addition, hPSC lines differentiate into three embryonic germ layers in vitro and in vivo and maintain a normal karyotype, confirming the pluripotency of these cells [1, 2, 25].

The present study demonstrated the beneficial effect of hUCS for culturing the supportive feeder cells of hPSCs. Notably, the important issues of using serum are pooling the serum from different donors and the lot-to-lot variability of hUCS. To control the quality of hUCS, several issues need to be considered such as the donors must not have any history of infectious diseases, the serum producing processes has to be carried following a strictly aseptic technique, or the serum has to be tested against contamination of endotoxins. Importantly, the ability of each batch of serum should be preliminary tested for culturing the fibroblast feeder cells prior to use the serum in a large scale. Alternatively, the HFF-hUCS conditions referred to in our study might be used for derivation of newly established hPSC lines prior to adaptation of such lines to the xeno-free/feeder-free culture conditions for the robust propagation of hPSC lines.

In conclusion, these results demonstrated that hUCS is an effective alternative source of serum for supplementation in the culture medium of HFFs. Not only does hUCS support the growth of HFFs, but inactivated HFF-hUCS also maintain the characteristics of supportive feeder cells for the culture of hPSC lines. These findings benefit the optimization of the xeno-free culture conditions of hPSCs. 

## 4. Materials and Methods

### 4.1. Human Subjects

In the present study, the use of human subjects, human cell lines, and serum was conducted in accordance with the guidelines and regulations of the Medical Council of Thailand and Faculty of Medicine, Chulalongkorn University.

### 4.2. Serum

Two types of serum were used in the present study, human umbilical cord blood-derived serum (hUCS) and fetal bovine serum (FBS). The hUCS was collected by our collaborator and the serum collecting protocol was described previously [16, 17]. In brief, each mother who donated the cord blood was monitored to ensure no history of infectious diseases such as hepatitis and human immunodeficiency virus (HIV). Cord blood serum was obtained by following blood clotting at room temperature for 6–12 hours and centrifugation at 2,800 rpm for 5 minutes at 20°C. The hUCS was filtered through the 0.22 um pore size. Twenty serum samples were pooled in each batch, tested for mycoplasma contamination, and kept at −20°C before being used. This hUCS is sufficient for ensuring growth and for maintaining the multipotency of human mesenchymal stem cells as described previously [16, 17]. HyClone FBS (recommended by the provider of human foreskin fibroblasts) was purchased from Thermo Fisher Scientific (Logan, UT, USA).

### 4.3. Culture of Human Foreskin Fibroblasts

Frozen human foreskin fibroblasts (HFFs; catalog number CRL 2429) were purchased from the American Type Culture Collection (ATCC; Manassas, VA, USA). CRL-2429 HFFs demonstrated to be a supportive feeder cells for the generation and culture of hESC lines [23–25]. The CRL-2429 HFFs were initially isolated and cultured in FBS-containing medium. HFFs at passage number (p) p4 were purchased from the supplier. Upon receiving the frozen stock, the cells were thawed and expanded according to the manufacturer's instructions with slight modifications. Briefly, the cells were cultured using HFF culture medium comprising 88% Iscove's modified Dulbecco's medium (IMDM; Thermo Scientific), 1% Glutamax (Invitrogen, Carlsbad, CA, USA), 1% penicillin-streptomycin (Invitrogen), and 10% FBS in 10 cm tissue culture dishes at 37°C in 5% CO_2_ atmosphere. At 80–90% confluency, the cells were dissociated with TrypLE Select (Invitrogen) and from p5 (p4 + 1) onward, HFFs were cultured in the medium containing either (i) 10% FBS or (ii) 10% hUCS. In addition, HFFs were cultured in the medium without serum supplementation and 10% knockout serum replacement (KSR; Invitrogen).

### 4.4. Proliferation of HFFs

The proliferation of HFFs was determined by calculating the population doubling time (PDT). The HFFs were cultured in hUCS-containing medium (HFF-hUCS) or FBS-containing medium (HFF-FBS), and the PDT was determined between p4 + 1 and p4 + 13. A total of 1 × 10^5^ cells were plated per one well of a 6-well dish. The cells were dissociated, counted, and passaged at 80–90% confluency, which occurred at approximately 4 days after replating. At each passage, the PDT was determined using the formula PDT = [log10(NH) − log10(N1)]/log10(2), where N1 is the plated cell number and NH is the cell number at harvest, as previously described [12]. The cell proliferation test was performed in triplicate.

### 4.5. Preparation of Feeder Cells

HFF-hUCS and HFF-FBS from p4 + 5 to p4 + 10 were used to prepare the feeder cells. HFFs were treated with 10 *μ*g/mL of mitomycin C (Sigma-Aldrich, St. Louis, MO, USA) for 3 hours. Treated HFFs were washed 5 times with PBS, dissociated with TrypLE Select (Invitrogen), and plated on the culture dishes at a density of 5 × 10^4^ cells/cm^2^.

### 4.6. Culture of Human Pluripotent Stem Cells

The karyotypically normal hESC line (Chula2.hES) was derived from frozen embryos [25] between p15 and p30, and the nonintegrating hiPSC line (HFSK#11.hiPS) was generated from human dermal fibroblasts using a Sendai virus carrying exogenous genes [36] between p15 and p30. Originally, both hPSC lines were derived and cultured on HFF feeder layer. The hPSC lines were continuously maintained on either mitomycin-C inactivated HFF-FBS or mitomycin-C inactivated HFF-hUCS in serum-free hPSC culture medium. The serum-free hPSC culture medium comprises 80% knockout Dulbecco's modified Eagles medium (KO-DMEM), 20% knockout serum replacement (KSR), 1% nonessential amino acid, 1% Glutamax, 1% penicillin-streptomycin, 0.1 mM *β*-mercaptoethanol (all from Invitrogen), and 8 ng/mL basic fibroblast growth factor (bFGF; R&D Systems, Minneapolis, MN, USA).

The culture medium was changed daily, and the hPSC lines were mechanically passaged using a 23 G needle every 5–7 days. After continuous culturing on inactivated HFF-FBS or HFF-hUCS for at least 10 passages, the expression of pluripotent markers was determined through immunostaining, RT-PCR, and flow cytometry. In addition, karyotypic analysis and in vitro and in vivo differentiation were performed.

### 4.7. Immunostaining for Pluripotent Markers

After continuous culturing for at least 10 passages on either inactivated HFF-FBS or HFF-hUCS, the expression of surface and intracellular markers, including stage-specific embryonic antigens (SSEA)-3, tumor-rejection antigen (TRA)-1-60, TRA-1-81, and transcriptional OCT-4, was determined. To detect the surface markers, hPSC colonies were fixed using 4% PFA for 15 minutes, washed 3 times with PBS, and incubated with blocking solution, containing 5% goat serum or 5% rabbit serum (Sigma) in PBS, for 1 hour. The fixed cells were incubated with primary antibodies at 4°C overnight. All primary antibodies were diluted 1 : 100 with blocking solution. After overnight incubation with primary antibodies, the cells were washed 3 times with PBS, followed by incubation with secondary antibodies for 1 hour at room temperature. The secondary antibodies were diluted at 1 : 200 with PBS. Negative controls were performed without the addition of primary antibodies. After incubation with secondary antibodies, the cells were washed 3 times with PBS and incubated for 10 minutes with 4′,6-diamidino-2-phenylindole (DAPI; Sigma) for nuclear staining. Subsequently, the cells were washed again and examined under using fluorescence microscopy. The primary antibodies used in the present study were SSEA-3 (Abcam, Cambridge, MA, USA), TRA-1-60 (Chemicon Millipore, Billerica, MA, USA), TRA-1-81 (Chemicon Millipore), OCT-4 (Abcam), NESTIN (Chemicon), PAX6 (Abcam), BRACHYURY (Abcam), smooth muscle actin (SMA; Abcam), alpha-fetoprotein (AFP; Abcam), and CDX2 (Abcam). The secondary antibodies used in the present study were FITC-conjugated goat anti-rabbit IgM (Abcam), FITC-conjugated goat anti-mouse IgM (Abcam), or Cy3-conjugated goat anti-mouse IgG (Chemicon).

### 4.8. Gene Expression Analysis

Reverse transcription polymerase chain reaction (RT-PCR) was performed for analyzing the expression of supportive feeder cell-related genes (Activin A, FGF2, TGF-*β*1, and BMP4) in inactivated HFF-FBS and inactivated HFF-hUCS and the expression of pluripotent-specific genes (OCT-4, NANOG, REX1, and UTF) in hPSCs cocultured with inactivated HFF-FBS or inactivated HFF-hUCS as well as the differentiated genes (NESTIN, BRACHYURY, and AFP) in embryoid bodies (EBs). The total RNA of each condition from three independent experiments was extracted and the expression of genes was analyzed.

Total RNA from EBs, hPSCs, inactivated HFF-FBS, and inactivated HFF-hUCS was extracted using GeneJet (Fermentas, Thermo Fisher Scientific, Baltics, Vilnius, Lithuania) according to the manufacturer's instructions. One microgram of total RNA was reverse transcribed to complementary DNA (cDNA) using the RevertAid H Minus First Strand cNDA Synthesis Kit (Fermentas, Thermo Fisher Scientific) according to the manufacturer's instructions. Polymerase chain reaction (PCR) was performed using the KAPA2G Fast HotStart ReadyMix (2x) (KAPABIOSYSTEMS, Cape Town, South Africa). PCR products were visualized in a 1% agarose gel. The PCR conditions and primers have been described elsewhere [2, 21].

### 4.9. Flow Cytometry

After coculture of hPSCs with inactivated HFF-FBS or inactivated HFF-hUCS feeder cells for at least 10 passages, the expression of SSEA-4 of hPSCs was evaluated using flow cytometry. At day 6 after passage, the hPSCs colonies were mechanically harvested and dissociated into single cells using TrypLE Select (Invitrogen), followed by centrifugation at 1500 rpm for 5 minutes. The cells were washed with PBS, centrifuged, and stained with FITC anti-human SSEA-4 (BioLeagent, San Diego, CA, USA) at 37°C for 1 hour. The cell nuclei were counterstained using propidium iodine (PI; Sigma). The cells were washed once in PBS, resuspended in 500 *μ*L of PBS, and subjected to analysis using flow cytometry (BD Bioscience, San Jose, CA, USA). The experiment of flow cytometric analysis has been repeated three times.

### 4.10. In Vitro Differentiation

To evaluate the hPSC differentiation in vitro, the hPSC colonies were cut into small clumps and cultured in hESC culture medium lacking bFGF to induce EB formation. EBs were cultured in suspension medium for 7 days, and subsequently plated on Matrigel-coated dishes for an additional 14 days. After culturing for a total of 21 days, the cells were immunostained. The differentiated cells were fixed, permeabilized, and immunostained as described above. Differentiated cells were immunostained for ectoderm (NESTIN, PAX6), mesoderm (BRACHYURY, SMA), endoderm (AFP), and trophoblasts (CDX2) markers. In addition, RT-PCR was performed for detection of differentiated gene expression of 21-day-old EBs as described above.

### 4.11. In Vivo Differentiation

The in vivo differentiation of hPSCs was examined by teratoma formation assay. The protocol of teratoma formation assay was similar to our previous report [25]. In brief, a total of 1 × 10^6^ cells of undifferentiated hPSCs were subcutaneously injected into the flank area of 6–8-week-old nude mice. Two mice were used for teratoma test of each group of hPSCs. The injected cells were allowed to differentiate for 8–10 weeks. After that, the mice were euthanized and the teratoma tissues were removed from the mice. The teratoma tissues were fixed with a 10% paraformaldehyde solution and embedded in paraffin block. The 4 *μ*m sections were stained with hematoxylin and eosin (H&E) and examined for embryonic germ layers. The care of immune-deficient mice was conducted in accordance with the institute guideline of the Ethical Committee for Animal Laboratory Use (Approval Number 7/57).

### 4.12. Karyotypic Analysis

To analyze chromosomal stability, the karyotype analysis of HFF-FBS and HFF-hUCS at p4 + 13 and hPSCs after continuous culturing on two different types of feeder cells for 15 passages was performed. HFF-FBS and HFF-hUSC were incubated with 0.1 *μ*g/mL of colcemid (Invitrogen) for 15 hours, while hPSCs were incubated for 3 hours. The cells were subsequently trypsinized and incubated in 0.075 M KCl for 20 minutes at 37°C in a 5% CO_2_ atmosphere, followed by fixing with 3 : 1 methanol/acetic acid. The metaphases were spread onto microscope slides and stained using a standard G banding technique. The chromosomes were classified according to the International System for Human Cytogenetic Nomenclature (ISCN).

### 4.13. Statistical Analysis

Statistical analyses were performed using SPSS software version 22.0. Student's* t*-test was used to evaluate differences in the rate of population doubling time and the percentage of SSEA-4 expression. The results were given as means ± standard error of mean (SEM), and values of *P* < 0.05 were considered statistically significant.
